# Management of traumatic brachial artery injuries: A report on 49 patients

**DOI:** 10.4103/0256-4947.51797

**Published:** 2009

**Authors:** Hasan Ekim, Mustafa Tuncer

**Affiliations:** a Department of Cardiovascular Surgery, and Yüzüncü Yil University, Van, Turkey; b Department of Cardiology, Yüzüncü Yil University, Van, Turkey

## Abstract

**BACKGROUND AND OBJECTIVE:**

The brachial artery is the most frequently injured artery in the upper extremity due to its vulnerability. The purpose of our study was to review our experience with brachial artery injuries over a 9-year period, describing the type of injury, surgical procedures, complications, and associated injuries.

**PATIENTS AND METHODS:**

Forty-nine patients with brachial artery injury underwent surgical repair procedures at our hospital, from the beginning of May 1999 to the end of June 2008. The brachial artery injuries were diagnosed by physical examination and Doppler ultrasonography. Depending on the mode of presentation, patients were either taken immediately to the operating room for bleeding control and vascular repair or were assessed by preoperative duplex ultrasonography.

**RESULTS:**

This study group consisted of 43 males and 6 females, ranging in age from 6 to 65 years with a mean (SD) age of 27.9 (6.7) years. The mechanism of trauma was penetrating in 45 patients and blunt in the remaining 4 patients. Stab injury was the most frequent form of penetrating trauma (24 of 45). Treatment included primary arterial repair in 5 cases, end-to-end anastomosis in 28 cases, interposition vein graft in 15 cases, and interposition-ringed polytetrafluoroethylene graft in 1 case. Associated injuries were common and included venous injury (14), bone fracture (5), and peripheral nerve injury (11). Fifteen patients developed postoperative complications. One patient underwent an above-elbow amputation.

**CONCLUSIONS:**

Prompt and appropriate management of the brachial artery injuries, attention to associated injuries, and a readiness to revise the vascular repair early in the event of failure will maximize patient survival and upper extremity salvage.

Upper extremity arterial trauma may significantly impact the outcome of the trauma patient, but the literature regarding this topic is scarce. Although relatively uncommon, injuries to the arteries of the upper extremity are serious and have the potential to significantly impact the outcome of the trauma patient.^1^ The brachial artery is the most frequently injured artery in the upper extremity. Its injury accounts for approximately 28% of all vascular injuries.^2^ The degree of ischemia after brachial artery injuries depends on whether the injury is proximal or distal to the profunda brachii.^3^

All brachial artery injuries can be managed successfully unless associated with severe concomitant damage to nerves. The median nerve courses with the brachial artery throughout its length. The radial and ulnar nerves parallel portions of the brachial artery. Therefore, as in all upper extremity vascular injuries, there is a high incidence of associated nerve injuries with brachial artery injuries.^3^ The purpose of our study was to review our experience with brachial artery injuries over a 9-year period. The type of injury, surgical procedures, complications, and associated injuries were reviewed.

## PATIENTS AND METHODS

**Figure 1:**
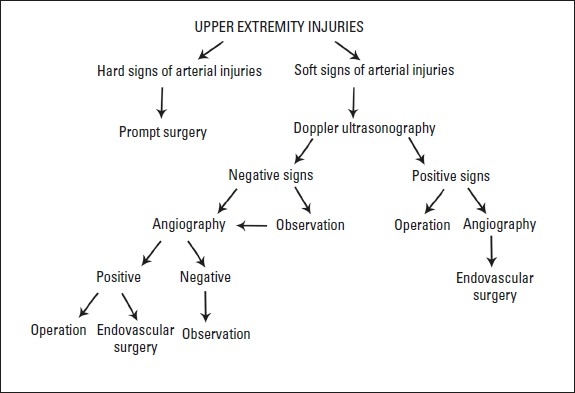
Diagnostic and therapeutic algorithm for upper extremity arterial injuries (soft signs include cool extremity, color change and nonexpanding hematoma).

Forty-nine patients with brachial artery injury underwent surgical repair procedures at our hospital from the beginning of May 1999 to the end of June 2008. The brachial artery injuries were diagnosed by physical examination and Doppler ultrasonography. The following findings were considered to be signs of arterial injury: brisk bleeding, expanding pulsatile hematoma, pale and cold upper extremities, absent or weak radial and ulnar pulses and associated profound neurological deficits. In this series, an average brachial-brachial Doppler index less than 0.5 was considered diagnostic for brachial artery injury. The initial management of the patients was conducted according to the principles of the advanced trauma and life support (ATLS) guidelines for trauma management.^4^ For patients presenting with hard signs of penetrating vascular injury, prompt surgical intervention without further diagnostic evaluation was used. Patients with blunt arterial injury or with penetrating injuries with clinical soft signs (cod extremity, color change, nonexpanding hematoma) underwent plain upper extremity radiography and Doppler ultrasonography (Figure 1).

The indications for fasciotomy were clinically evident or impending compartment syndrome, massive swelling in the upper limb, ischemia lasting more than 6 hours and any motor or sensory deficits. In all patients with associated bone fracture, vascular repair always preceded orthopedic reconstruction. Endoluminal shunts were not required in any of the patients. Successful repair was assessed by the return of radial and ulnar pulses at the end of the operation.

Patients with more severe soft tissue and muscle injuries were treated with thorough debridement of all grossly nonviable tissue, with removal of foreign bodies and copious irrigation with isotonic saline solution, and then injured vessels were exposed. Heparin was administered intravenously for systemic anticoagulation before the vessels were clamped proximally and distally with nontraumatic vascular clamps. Fogarty catheters were used for thrombectomy of the distal and proximal arterial segments. All patients received intravenous heparin for a period of 5-7 days postoperatively and were discharged home on oral aspirin 100 mg/day for a period of 3 months.

**Figure 2:**
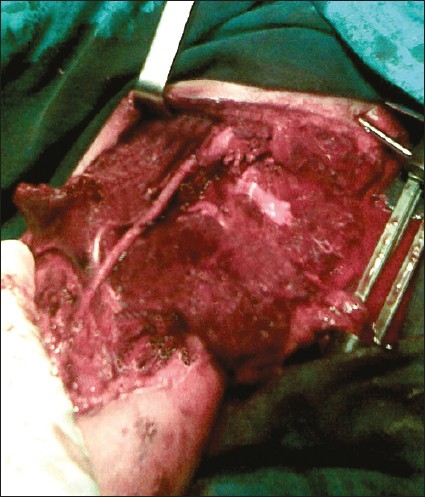
Reconstruction of crushed brachial artery with saphenous vein interposition graft.

Exposure of the brachial artery in the arm was always approached with a median incision in the line of the sulcus separating the biceps muscle from the triceps muscle. The median nerve was always identified and separated from the brachial artery. Exposure of the artery at the elbow was performed with a skin crease incision made at the elbow, with longitudinal extensions along the line of the brachial artery medially and down the brachioradialis laterally. Primary arterial repair or end-to-end anastomosis was preferred whenever possible; otherwise, the interposition saphenous vein graft was used (Figure 2).

All associated brachial venous injuries except one were repaired, in an attempt to prevent postoperative venous hypertension and to minimize development of compartment syndrome. Associated nerve injuries were repaired whenever possible. Repaired vessels, especially at the anastomotic suture lines and graft location, were compulsorily covered with muscles and soft tissue to prevent desiccation and disruption. All patients received intravenous prophylactic antibiotics, which were continued postoperatively for 3 to 5 days, unless prolonged use was dictated by the presence of contamination or infection.

One month after hospital discharge, patients were routinely examined in the outpatient department where segmental pressures were measured and functional status of the upper extremity assessed. Thereafter, they were followed at 3-month periods.

Statistical analysis was performed using the paired samples t test to determine whether there was any statistically significant difference between preoperative and postoperative Doppler pressure indices. A *P* value of less than .05 was taken to indicate significance.

## RESULTS

This study group consisted of 43 males and 6 females, ranging in age from 6 to 65 years with a mean (SD) age of 27.9 (6.7) years. The right upper limb was the more frequently affected side as it was involved in 28 patients and the left side in 21 patients. The mechanism of trauma was penetrating in 45 patients and blunt in the remaining 4 patients (from road traffic accidents). Stab injury was the most frequent form of penetrating trauma (24 of 45). Other forms of penetrating trauma in a descending order of frequency were window glass injuries in 11 patients, gunshot injuries in 9 patients, and industrial accident in 1 patient. Thirty-four patients presented with hemorrhage, 28 with ischemia, 8 with hematoma and 1 with a pseudoaneurysm (Table 1).

**Table 1:** Signs, symptoms, and associated injuries among 49 patients with brachial artery injury.

Presentation	No. of patients
Pulse deficit	43
Pulse insufficiency	6
Hemorrhage	34
Hypotension	20
Peripheral nerve injury	11
Venous injury	14
Tendinous injury	8
Hematoma	8
Bone fracture	5
Pseudoaneurysm	1
Ischemia	28

In 42 patients the diagnosis of arterial injury was based on clinical and hand-held Doppler examination. Preoperative duplex scan was used in only 7 patients. Physical examination and Doppler ultrasonography revealed the absence of arterial pulses in 43 patients and weak arterial pulses in 6. For brachial artery injuries the average brachial-brachial Doppler pressure index was 0.434 (0.046), (range, 0.23 to 0.48) preoperatively, and 0.894 (0.031) (range, 0.83 to 0.93) postoperatively *(P<.05).* Treatment included primary arterial repair in 5 cases, end-to-end anastomosis in 28 cases, interposition vein graft in 15 cases and interposition ringed PTFE graft in 1 case. There were 14 patients with associated brachial vein injury, of which 4 cases had primary repair, 8 had end-to-end anastomosis and 1 had saphenous vein graft interposition. One severely injured brachial vein was compulsory ligated. Five of the 49 patients had injuries to bony structures. Fractures occurred most frequently among patients with blunt trauma (3 patients); fracture was also detected in 2 patients with gunshot wound. All fractures were treated with external fixation.

Eight patients had tendinous injuries that were repaired perioperatively by orthopedic surgeons. Additionally, fasciotomy was performed in 6 patients.

Eleven of 49 patients had peripheral nerve injury: 2 had injuries to the median and ulnar nerves, 6 had injuries to the median nerve alone and 3 had injuries to the ulnar nerve alone. Eight of the 11 nerve injury cases were primarily repaired perioperatively by neurosurgeons. Postoperatively, all patients with nerve injury underwent electromyelography for evaluation of nerve deficit. During the follow-up period, functional recovery was achieved in 6 of these patients. In the remaining patients, functional disability was evident throughout follow-up period. They were followed up neurosurgery and rehabilitation clinics.

Fifteen patients developed postoperative complications consisting of wound infection (2 patients), permanent nerve damage (4 patients), ischemic symptoms due to graft thrombosis (4 patients) and thrombosis of the venous repair (5 patients). Amputation was required in one patient who was involved in a traffic accident and suffered from a proximal brachial artery laceration with injuries to the brachial vein, and the median and ulnar nerves above the elbow in association with a fracture of the humerus. He was operated on 10 hours after injury. Arterial flow was reestablished successfully with a saphenous vein bypass graft and the brachial vein was ligated, but subsequent above-elbow amputation was required due the severity of concomitant nerve and bony injuries with a nonfunctional right upper limb and infection. Two patients with blunt trauma experienced wound infections, but these infections were resolved within 15 days by antibiotic therapy. Four patients experienced early postoperative graft thrombosis. In these patients, thrombectomy was performed. It was successful in three patients. Although thrombectomy was unsuccessful in the remaining one patient with prosthetic graft interposition, amputation of the limb was not required due to adequate collateral circulation.

Postoperatively, all patients who had brachial vein injury experienced various degrees of edema in the upper extremity. This edema decreased with elevation in all patients during the follow-up period (approximately 15 to 45 days). Postoperative venous Doppler studies showed thrombosed repair in five cases without any complication. No patients expired and 48 patients were discharged home with good functional vascular status and limitations based on the degree of associated nerve injury.

The average follow-up period was 20 months (range 3-32 months); follow-up visits were usually necessary for neurological examination.

## DISCUSSION

Penetrating trauma is generally considered to be the most common cause of a vascular injury in the upper extremity.^5^ In addition to the usual types of blunt and penetrating injuries, supracondylar fractures or dislocation of the humerus may injure the brachial artery.^3^

The role of angiography in patients with brachial artery injury appears to be controversial.^9^ Upper extremity arterial injury often can be managed without arteriography, particularly in cases with penetrating trauma^6^ as seen in this series. Bynoe et al^7^ has reported 99% sensitivity and 98% accuracy of duplex ultrasonography. Furthermore, duplex ultrasonography has no interventional risks and is more cost-effective for screening such injuries than angiography. Doppler ultrasonography was found to be more sensitive than angiography in an experimental trial, thereby supporting its use in the trauma setting.^8^ Doppler ultrasonography of the upper extremity has been shown to be as specific and sensitive as arteriography in detecting brachial artery injuries.^9^ If uncertainty remains regarding vascular injuries after physical examination and Doppler ultrasonography, angiography may be performed to confirm vascular injury.^9^

The mainstay of diagnosis of brachial artery injury in our study was based on clinical assessment and hand-held Doppler examination. Doppler ultrasonography was carried out in stable patients with associated soft signs of injury, to make a conclusive decision. We believe that angiography remains an effective method for diagnosing the vascular lesions, but it is also a time-consuming procedure, especially in traumatic vascular injuries requiring prompt surgery. Additionally, preoperative angiography did not offer any benefits in patients with obvious arterial injury. Angiography may be useful, especially in patients with multiple sites of potential vascular injury.^10^ We have used angiography in patients with stable axillary artery injury associated with chest trauma causing subcutaneous emphysema, which prevented ultrasonographic examination. Additionally, angiography must be used during endovascular surgery.

Normally the average brachial-brachial Doppler pressure index between the 2 upper extremities is approximately 0.95; it is rarely less than 0.85.^11^ In our serious, preoperative values were significantly lower than normal, but postoperative values were similar to normal. Duplex ultrasonography is fairly reliable, except with minor injuries, depending on the experience of the sonographers.^2^ However, in addition to assessing the upper extremity for pulse pressures by physical examination and continuous-wave Doppler, neuromuscular function, soft tissue involvement, and skeletal integrity should be evaluated.^1^

The timing of vascular repair in relation to fracture management has long been a source of controversy. Because prevention of prolonged tissue ischemia is the objective, the standard recommendation is for vascular repair to precede fracture management.^12^ In contrast, Hunt et al^13^ suggested that arterial revascularization should be followed by skeletal stabilization and nerve and tendon repair. In the rare instance when external fixation is immediately required to stabilize the limb following massive musculoskeletal trauma, temporary selective use of shunts to restore circulation may be used to allow rapid fixator placement, with later vascular and orthopedic repair.^1^ Unstable fractures jeopardize arterial repairs more in the upper extremities than in the lower extremities because of less adjacent muscle.^14^ Therefore, we have routinely controlled the repaired vessels after bone fixation for patency.

Surgical repair of brachial artery injuries can be accomplished by a variety of techniques, including lateral repair, resection with end-to-end anastomosis, or interposition grafting, usually with a saphenous vein.^3^ End-to-end anastomosis is preferable if it can be performed without tension or damage to major collateral vessels. Otherwise, the saphenous vein interposition graft is the next best choice, because it has better patency rates and better resistance to infection compared with synthetic grafts.^15^ However, we had to use a ringed polytetra-fluoroethylene (PTFE) graft in one patient with inadequate saphenous vein. Endovascular techniques have increasingly been used in the management of penetrating injuries and may have some advantages even in blunt trauma.^16^ They are especially ideal for managing blunt axillary artery injuries that are anatomically difficult to repair.^1^

It is important to limit the period of ischemia, and so minimize the degree of ischemia-reperfusion injury and the systemic consequences after the arterial repair. The extent of ischemia-reperfusion injury is directly proportional to the severity and duration of striated muscle ischemia. Beyond a golden period of 6 to 8 hours of ischemia, ischemia-reperfusion injury will endanger the viability of the limb and sometimes even the patient's life.^2^ In this series, while 9 patients experienced a duration of ischemia longer than this period, there was only one amputation. The infrequent need for amputation was probably related to the rich collateral circulation in the upper limb of most patients.^2^,^17^ Therefore, we suggest that all patients without obvious necrotic changes should be operated on irrespective of time interval between the beginning of trauma and arrival to the operating room, as was done in this series.

Neurological injury continues to destroy the function of the upper extremity even after a successful arterial repair.^9^ The rate of functional disability ranges from 27% to 44% when injury to the upper extremity includes nerve injuries.^18^ Nichols and Lillehei^19^ recommend primary nerve repair for penetrating trauma (lacerations and stab wounds), while with gunshot wounds, because of the degree of contusion, acute nerve repair is rarely indicated.^20^ Additionally, major venous injuries, fractures and widespread tissue destruction may also influence the long term function of the extremity.^21^

Venous injuries generally remain unrecognized until surgical exposure. The indication for venous repair in the upper limb is not clear. Injuries of the brachial or arm veins can be treated by ligation, as edema is rare. However, in the case of a severe soft-tissue injury where maximal venous return is necessary, venous repair is probably warranted.^5^ We would add that brachial venous continuity, when possible, is maintained.

In conclusion, the elbow, like the shoulder, is known to have extensive collateral circulation that may mask the signs of acute arterial injury,^22^ but whether this circulation is adequate is debatable.^23^ Therefore, all brachial artery injuries should be repaired. Careful clinical examination, Doppler ultrasonography and pressure measurements are as important as angiography in the diagnosis of vascular injuries.^9^ Also, the superficial location of brachial artery makes the diagnosis relatively simple. Therefore, brachial artery injuries may be diagnosed without angiography. We believe that angiography should be performed when the vascular injuries would be impossible to diagnose with other diagnostic modalities or when endovascular procedures are required. Prompt and appropriate management of the brachial artery injuries, attention to associated injuries and a readiness to revise the vascular repair early in the event of failure will maximize patient survival and upper extremity salvage.
